# Albumin redox status in the context of kidney transplantation – an observational, pilot study

**DOI:** 10.1186/s12882-026-05316-y

**Published:** 2026-08-28

**Authors:** Lea Marie Berntsen, Margret Paar, Katja Waterstradt, Kerstin Schnurr, Gerd Klinkmann, Steffen Mitzner, Karl Oettl, Andreas Kribben, Kristina Boss

**Affiliations:** 1 Former Department of Nephrology, University Hospital Essen, University Duisburg-Essen, Essen, Germany; 2 Department of Infectious Diseases and Nephrology, University Hospital Essen, University Duisburg-Essen, Essen, 45147 Germany; 3 Division of Medicinal Chemistry, Otto Loewi Research Center, Medical University of Graz, Graz, Austria; 4 MedInnovation GmbH, Berlin, Germany; 5 Department of Anaesthesiology and Intensive Care Medicine, University of Rostock, Rostock, Germany; 6 Department of Extracorporeal Immunomodulation, Fraunhofer Institute for Cell Therapy and Immunology, Rostock, Germany; 7 Department of Medicine, Division of Nephrology, University of Rostock, Rostock, Germany

**Keywords:** Albumin, Redox state, Kidney transplantation, Human nonmercaptalbumin

## Abstract

**Background and hypothesis:**

The albumin redox status (ARS) is altered in patients with chronic kidney disease (CKD), shifting towards a higher degree of oxidation. Oxidative Stress leads to persistent microinflammation and increased morbidity and mortality. An improvement in renal function following kidney transplantation (KTX) could lead to an improvement in ARS. Aim of this study was to evaluate the effects of KTX on ARS.

**Methods:**

In an observational, monocentric feasibility study design, ARS was determined before and eight times up to 180 days after KTX by fractionation of albumin into reduced human mercaptalbumin (HMA), reversibly oxidized human nonmercaptalbumin 1 (HNA-1) and irreversibly oxidized human nonmercaptalbumin 2 (HNA-2) by high-performance liquid chromatography (HPLC) with fluorescence detection. In healthy individuals, HMA accounts for 70–80%, HNA-1 for 20–30% and HNA-2 for about 2–5% of total albumin.

**Results:**

42 patients (62% male, median age of 43.5 years) were included in the study. Before KTX, HMA was reduced (median 63.5%, interquartile range [IQR] 59.6–67.3%), while HNA-1 (28.9%, IQR 25.9–33.2%) and HNA-2 (7.1%, IQR 6.0-8.1%) were elevated compared to a healthy population. Within the first week post-KTX, HMA declined further (57.9%, IQR 53.1–63.6%) and HNA-2 peaked (9.2%, IQR 7.3–11.4%). In most patients, the ARS returned to pre-KTX level by day 60. At day 180, 31% of patients still showed HNA-1 and HNA-2 levels above the respective reference ranges reported for healthy individuals, despite substantial recovery of kidney function.

**Conclusion:**

While kidney function was restored rapidly after kidney transplantation, ARS followed a slower and more variable recovery pattern, suggesting that factors beyond renal function may influence albumin redox state. Further research is needed to determine its clinical relevance and potential implications for transplant care.

**Supplementary Information:**

The online version contains supplementary material available at https://doi.org/10.1186/s12882-026-05316-y.

## Introduction

A key pathophysiological aspect in CKD patients is the qualitative alteration of albumin. Chronic functional impairment of albumin is accompanied by a shift in its redox status toward a higher degree of oxidation. This contributes to the increased morbidity and mortality in CKD patients, particularly in those with ESKD [1–4]. Chronically increased levels of inflammation and oxidative stress, further exacerbated by the uremic environment in ESKD patients, lead to secondary diseases such as atherosclerosis or hypertension and promote cardiovascular events [5, 6].

One of the mechanisms to counteract the action of reactive species is provided by antioxidant systems, including albumin. Its redox sensitive thiol in cysteine-34 (Cys-34) comprises the largest pool of extracellular thiols [7, 8]. The albumin redox state (ARS) can be defined in different ways. Here we understand it based on Cys-34, which may occur in three different states: (i) in the fully reduced form as a thiol, human mercaptalbumin (HMA), (ii) in a mildly and reversibly oxidized form as a disulfide mainly with another cysteine, human nonmercaptalbumin-1 (HNA-1) and (iii) irreversibly oxidized to the sulfinic or sulfonic acid form, human nonmercaptalbumin-2 (HNA-2) [8].

The degree of albumin modification has an impact on its functional properties and is associated with major endpoints [9]: increased levels of oxidized albumin correlate positively with increased mortality and cardiovascular events. Even in pre-dialysis phase, HNA-1 and HNA-2 increase as kidney function declines [10]. These findings suggest that ARS reflects, at least in part, the systemic oxidative environment associated with impaired kidney function. On the other hand, KTX has been demonstrated to enhance renal function and thereby directly eliminates pro-inflammatory uremic toxins. Consequently, pro-inflammatory markers such as CRP, IL-6 and TNF-α are reduced and endothelial function is enhanced, resulting in an overall lower level of oxidative stress [11]. Thus, restoration of renal function following KTX may also be expected to affect the redox state of circulating albumin. Whether these changes are reflected in ARS after KTX, however, remains unclear.

Given the association of albumin oxidation with systemic oxidative stress and adverse clinical outcomes in CKD, changes in ARS following KTX may provide insight into the pathophysiological consequences of restoration of renal function. However, there is currently a knowledge gap regarding whether, and to what extent, KTX affects ARS and which clinical parameters may be associated with alterations in ARS. Therefore, this observational pilot study aimed to investigate the extent to which ARS is influenced by KTX and to explore parameters associated with changes in ARS. The present study was designed as an exploratory first step and was not intended to establish ARS as a prognostic biomarker.

## Materials and methods

### Study design and patient enrolment

The study was conducted in the former Department of Nephrology at the University Hospital Essen in Essen, Germany in an observational, monocentric design. Since this is a pilot study—in the sense of an initial, purely exploratory feasibility study—no power analysis was conducted prior to the study. All patients who received a kidney transplant, fulfilled the inclusion criteria and gave written informed consent were included in the study from February 2023 to May 2024. The inclusion criteria were age of at least 18 years and the ability to give written informed consent. The exclusion criteria were underage and lack of written consent. Patients on haemo- and peritoneal dialysis were included, as well as patients receiving living or deceased donation and patients transplanted pre-emptively. The baseline data set was collected immediately before KTX and regarded as pre-dialysis data. Eight more study visits were performed at day 2, 4, 7, 14, 30, 60, 90 and 180 after KTX.

### Baseline laboratory measurements

Baseline laboratory measurements were performed on each visit by the Central Laboratory of the University Hospital Essen, Essen, Germany. Albumin quantity was determined using Albumin Bromocresol Green (BCG) Assay Kit (Abcam) by UV-Vis spectrometry. Glomerular filtration rate (GFR) was estimated by using the CKD-EPI formula 2012 [6] including Cystatin C and enzymatic creatinine. Hs-CRP was measured using the turbidimetry Atellica CH by Siemens Healthineers AG, Eschborn, Germany. Interleukin-6 was measured using Sequential Solid-Phase Chemiluminescent Immunoassay.

### Determination of albumin redox state

The albumin redox state (ARS) was assessed by separating albumin into its fractions—human mercaptalbumin (HMA), human nonmercaptalbumin-1 (HNA-1), and human nonmercaptalbumin-2 (HNA-2)—using high-performance liquid chromatography (HPLC) according to the method described by Oettl et al. [8]. The interpretation of the study findings is based on the relative proportions of these albumin redox fractions. Within the scope of this study, no measurements of ARS proportions were performed in healthy individuals. Absolute concentrations of HMA, HNA-1, and HNA-2 were obtained by multiplying the corresponding fraction by the serum albumin concentration (g/dL).

### Statistical analysis

The study population was described using frequency distribution and measure of central tendency and variability. The Kolmogorov-Smirnov test was used for normality testing and excluded normality for some data sets. Residual Maximum Likelihood as a linear mixed-effects model (LMMs) with Geisser-Greenhouse-Correction was performed to determine whether there were statistically significant differences in the measured values across the different time points. Statistical significance was set at the level of *p* < 0.05. Post-hoc analyses were performed by Tukey-test and Tukey-Kramer-test in case of unequal sample sizes. All statistical analyses and graphical evaluations were performed with GraphPad Prism software by Domatics (version 10.1.2 San Diego, CA, USA) and Microsoft 365 Excel (version 2409).

### Ethics approval

The study was performed in accordance with the Declaration of Helsinki and the International Conference on Harmonization Good Clinical Practice guidelines. The study was approved by the local Ethics Committee of the University of Duisburg-Essen (Ethics No.: 22-10806-BO).

## Results

### Patient and treatment characteristics

A total of 42 patients (62% male) with a median age of 43.5 years (range: 18–68 years) were included in this study. Six patients received thymoglobulin as induction therapy; all other patients received basiliximab. Maintenance therapy for all patients consisted of prednisolone, tacrolimus, and mycophenolate mofetil. Infectious diseases occurred in 18 patients, predominantly urinary tract infections within the first 14 days after KTX. These were treated with antibiotics. Delayed graft function (DGF) occurred in four patients (one male and three female patients; median age: 56.5 years, range: 38–64 years) and was characterized by temporary anuria or the requirement for temporary dialysis following kidney transplantation (KTX). One of these cases was associated with rejection. All four patients had undergone haemodialysis for at least seven years prior to KTX and received grafts from deceased donors.

One female patient experienced primary non-function due to rejection but remained part of the study cohort, as dialysis treatment was not required.

Biopsy-confirmed rejection episodes were observed in six patients and classified according to the 2022 Banff Classification for Renal Transplant Pathology. Detailed patient characteristics are summarized in Table 1 and Supplement Tables 4 and 5.

**Fig. 1:**
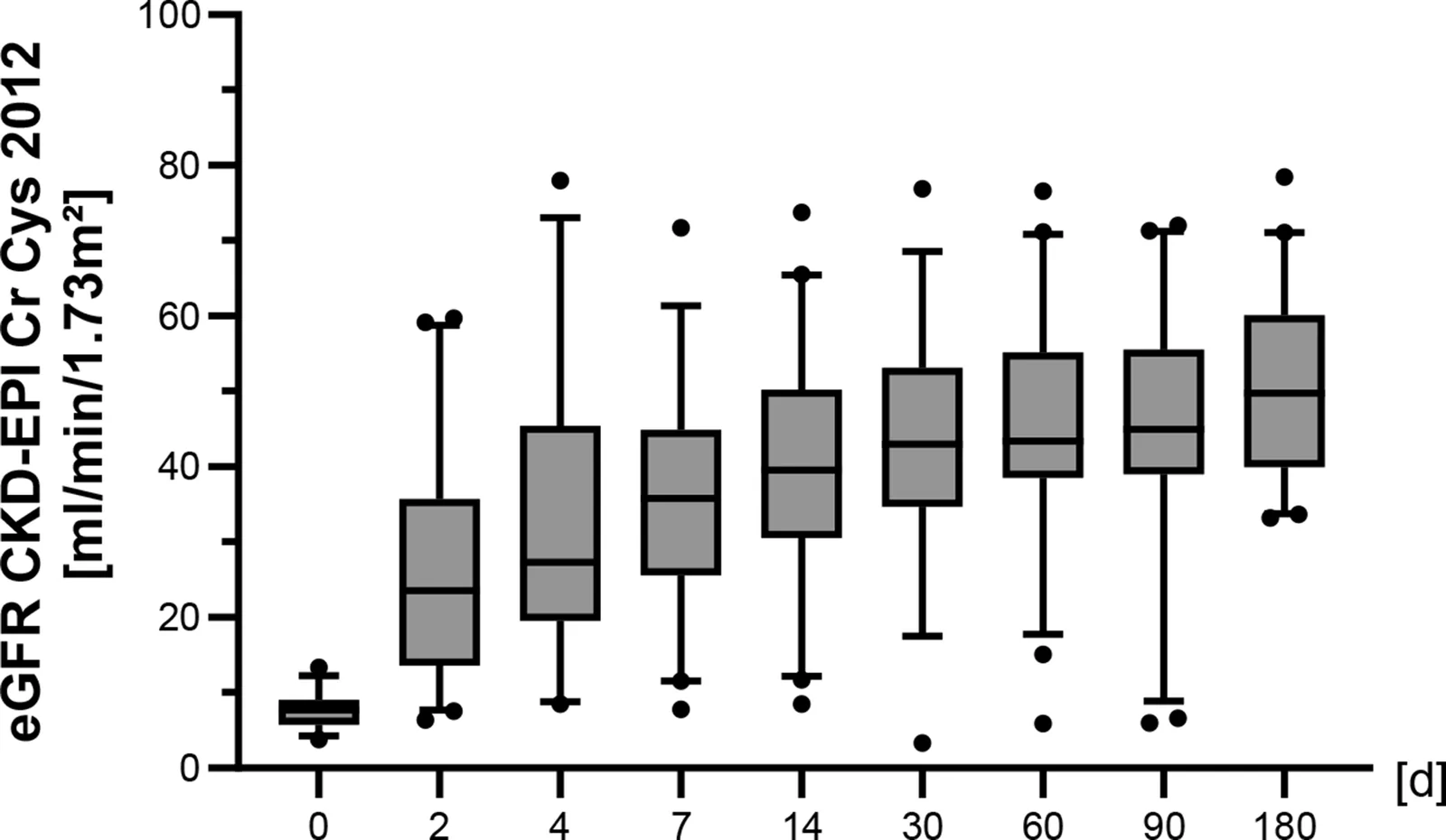
Estimated glomerular filtration rate after kidney transplantation. Shows the eGFR at 0 to 180 days after KTX with *n* = 42 patients measured by the eGFR CKD-EPI formula, including serum-creatinine and cystatin-C; the centre line indicates the median, the upper and lower limits represent 25th and 75th percentiles, the whiskers the 5th and 95th percentiles. Highest and lowest values are shown as dots

Following KTX, renal function improved rapidly and stabilized by day 90, with a median eGFR of approximately 45 mL/min/1.73 m² (Fig. 1). At 180 days post-transplantation, 38.1% of patients were classified as having CKD stage 3a, 33.3% as stage 3b, and 23.8% as stage 2.

Serum albumin levels decreased from a baseline median of approximately 4.5 g/dL (IQR 4.1–4.7 g/dL; Supplementary Table 2) to a nadir of 3.3 g/dL (IQR 3.2–3.6 g/dL) on day 4 after transplantation. Albumin levels subsequently recovered progressively from day 7 onward, returning to pre-transplant values by day 90 (median 4.5 g/dL, IQR 4.3–4.8 g/dL) and remaining stable at day 180 (median 4.6 g/dL, IQR 4.3–4.8 g/dL). None of the patients received intravenous commercial albumin or high protein dietary. Detailed laboratory data, including longitudinal changes in serum albumin levels and kidney function, are provided in Supplementary Tables 1 and 2.

**Table 1:** Patient characteristics

Characteristic	All *n* = 42	Deceased donation *n* = 28	Living Donation *n* = 14
**Demographic data**			
Age [years], median (range)	43.5 (18–68)	48.5 (40.3–57.5)	29.5 (23.5–40)
Men, n [%]	26 [62]	17 [61]	9 [64]
Women, n [%]	16 [38]	11 [39]	5 [36]
**Comorbidities, n [%]**			
Liver disease			
• Steatosis hepatis I° • Chronical hepatitis	2 [5] 4 [10]	2 [7] 4 [14]	0 0
Diabetes mellitus type 2	1 [2]	1 [4]	0
Cardiovascular disease			
• Heart transplanted • Heart failure • Valve diseases • Hypertension • Peripheral arterial disease • Atrial fibrillation • Coronary heart disease	1 [2] 3 [7] 7 [17] 32 [76] 2 [5] 4 [10] 4 [10]	1 [4] 2 [7] 5 [18] 21 [75] 2 [7] 3 [11] 3 [11]	0 1 [7] 2 [14] 11 [79] 0 1 [7] 1 [7]
**Infectious complications, n [%]**			
• Urinary tract infections • Bloodstream infection • Sepsis • COVID-19	18 [43] 4 [10] 1 [2] 1 [2]	14 [50] 2 [7] 1 [4] 1 [4]	4 [29] 2 [14] 0 0
**Renal disease, n [%]**			
Glomerulonephritis (others than IgA nephritis)	13 [31]	7 [25]	6 [43]
IgA nephritis	12 [29]	7 [25]	5 [36]
ADPKD	4 [10]	4 [14]	0
Diabetic nephropathy	1 [2]	1 [4]	0
Hypertensive nephropathy	1 [2]	1 [4]	0
Other (aHUS, Alport, SLE)	9 [21]	6 [21]	3 [21]
Unknown aetiology	2 [5]	2 [7]	0
Dialysis *			
Time [months], median (range)	72 (0-168)	97.4 (22–168)	8.5 (0–27)
Haemodialysis, n [%]	31 [74]	19 [68%]	9 [64]
• Men / women, n	18 / 13	11 / 8	5 / 4
• Age [years], median (range)	43.5 (33-56.3)	49.5 (40-58.5)	31 (25–37)
Peritoneal dialysis, n [%]	8 [19]	6 [21]	2 [14]
• Men / women, n	6 / 2	4 / 2	2 / 0
• Age [years], median (range)	46 (36.5–51.8)	46 (42-49.3)	38.5 (29.3–47.8)
**Pre-emptive, n**	3	0	3
Men/women, n	2 / 1		2 / 1
Age [years] median (range)	25 / 23-26.5		25 (23-26.5)
**Surgery data**			
Warm ischemia time [minutes] Median (range)	23 (18–29)	25 (20–32)	18.5 (16-24.3)
Cold ischemia time [minutes] Median (range)	492 (166–775)	716 (492–846)	154 (136–169)
**Dysfunctions, n**			
Delayed graft function Primary non-function Rejections	4 1 6	4 1 5	0 0 1
• TCMR (Banff category 4) • ABMR (Banff category 2) • Borderline (Banff category 3)	3 1 2	2 1 2	1 0 0

Patient characteristics include age, gender, renal underlaying disease, dialysis before KTX, comorbidities including diabetes mellitus diagnosed before KTX, number of pre-emptive transplanted patients, information about ischemic times and clinical data. *For description of dialysis data, patients who received KTX pre-emptively were excluded of the group “living donation”, which makes *n* = 11 in this column

Abbreviation: antibody mediated rejection (ABMR), Autosomal dominant polycystic kidney disease (ADPKD), atypical haemolytic uremic syndrome (aHUS), immunoglobulin A (IgA), Haemoglobin A1c (HbA1c), Systemic lupus erythematodus (SLE), T-cell mediated rejection (TCMR)

### Albumin redox state

Before KTX, HMA levels were more than 15% points lower than those reported for healthy individuals [8], accompanied by increased oxidized fractions (HMA median 63.5% [IQR 60.0–67.2%], HNA-1 28.9% [26.0–33.4%], HNA-2 7.1% [6.3–8.2%]). Following KTX, the ARS changed further. Within the first postoperative week, HMA decreased significantly to a nadir of 57.9% (53.1–63.6%, *p* = 0.0019), whereas oxidized albumin fractions peaked (HNA-1: 32.6% [28.0–35.2%]; HNA-2: 9.2% [7.3–11.4%]). Acute rejections occurred in six patients in the early period after KTX and were associated with HNA-2 values up to 17.1%. In addition, one patient underwent plasmapheresis with 4 L fresh-frozen plasma, resulting in an HNA-2 outlier of 22% (0.55 g/dL) one day after treatment.

By day 60 after KTX, HMA had returned to baseline and increased slightly thereafter up to day 180. HNA-1 remained mildly elevated during the first two months before declining to 28.9% (IQR 24.7–31.7%) or 1.26 g/dL (IQR 1.15–1.44 g/dL) at day 180. In contrast, HNA-2 decreased continuously after the immediate postoperative rise and reached a minimum of 5.6% (IQR 5.3–6.2%) at day 180. Kidney function improved rapidly after KTX, with most patients reaching CKD stage 3a or better by day 90. Before KTX, hs-CRP and IL-6 were within the normal range. In the first postoperative days, both parameters were slightly elevated. However, unlike HNA-1 and HNA-2, hs-CRP and IL-6 returned to the normal range from day 30 through day 180 after KTX. HNA-1 and HNA-2 showed a much slower decline.

Marked perioperative albumin loss significantly affected the absolute concentrations of albumin redox fractions. HMA decreased from 2.62 g/dL preoperatively to 1.85 g/dL (IQR 1.65–2.08 g/dL) within one week after KTX (*p* = 0.009) and recovered to 2.08 g/dL by day 60 (*p* = 0.0049). Although the proportion of HNA-1 increased slightly, its concentration declined from 1.26 g/dL to a nadir of 0.91 g/dL on day 2 before returning to baseline by day 60 (*p* < 0.0001). Despite the overall reduction in albumin levels, HNA-2 concentrations remained stable during the first postoperative week and subsequently declined continuously until day 180 (*p* = 0.0004).

Women exhibited greater ARS alterations than men at most time points. On day 7 after KTX, median HMA was 56.3% (IQR 56.3–59.3%) in women and 60.3% (IQR 54.0–62.2%) in men, whereas HNA-2 was 11.1% (IQR 7.4–14.5%) and 7.9% (IQR 7.2–10.4%), respectively. These trends persisted at day 180, although none reached statistical significance (lowest p-value 0.18).

Recipients of deceased-donor kidneys consistently showed higher HNA-2 fractions than recipients of living-donor organs. In deceased-donor recipients, HNA-2 increased more markedly early after transplantation, reaching 9.4% (IQR 8.0–10.5%) on day 2 versus 7.5% (IQR 7.0–8.5%) in living-donor recipients (*p* = 0.03), and peaking at 9.8% (IQR 9.0–11.6%) on day 4 versus 7.7% (IQR 7.9–8.7%; *p* = 0.002). These differences remained up to 180 days after KTX, though this was not statistically significant (lowest p-value 0.08 at day 14).

**Fig. 2:**
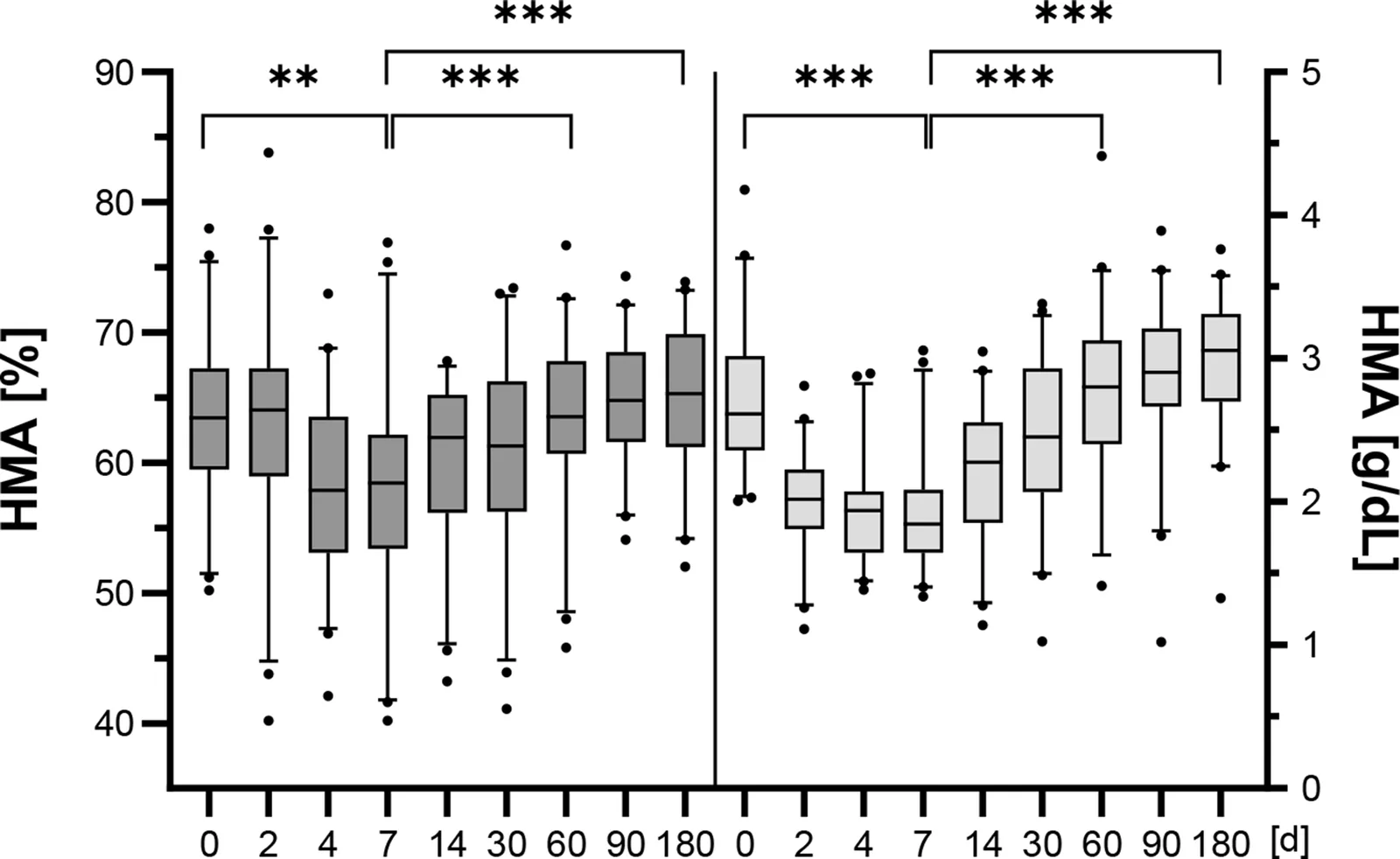
Human mercaptalbumin after kidney transplantation. Shows the changes in human mercaptalbumin (HMA) at 0 to 180 days after KTX with *n* = 42 patients in percentage (left y-axis) and total amount (right y-axis); the centre line indicates the median, the upper and lower limits represent the 25th and 75th percentiles, the whiskers the 5th and 95th percentiles. Highest and lowest values are shown as dots. Asterisks mark differences between time points at a significance level of ** = *p* < 0.01 and *** = *p* < 0.001 with Residual Maximum Likelihood as a linear mixed-effects model (LMMs) and post-hoc Tukey-Kramer test. Additional significances could be shown between day 0 and 4 (*p* = 0.0047), 0 and 14 (*p* = 0.0400), 2 and 4 (*p* = 0.0270), 2 and 7 (*p* = 0.0062), 4 and 60 (*p* = 0.0053), 4 and 90 and 180 (each *p* < 0.0001), 7 and 90 (*p* < 0.0001), 14 and 60 (*p* = 0.0339), 14 and 90 and 180 (each *p* < 0.0001), 30 and 90 (*p* = 0.0005) and 30 and 180 (*p* = 0.0065) in percentage HMA. These further significances were not included in the figure for a clearer readability

**Fig. 3:**
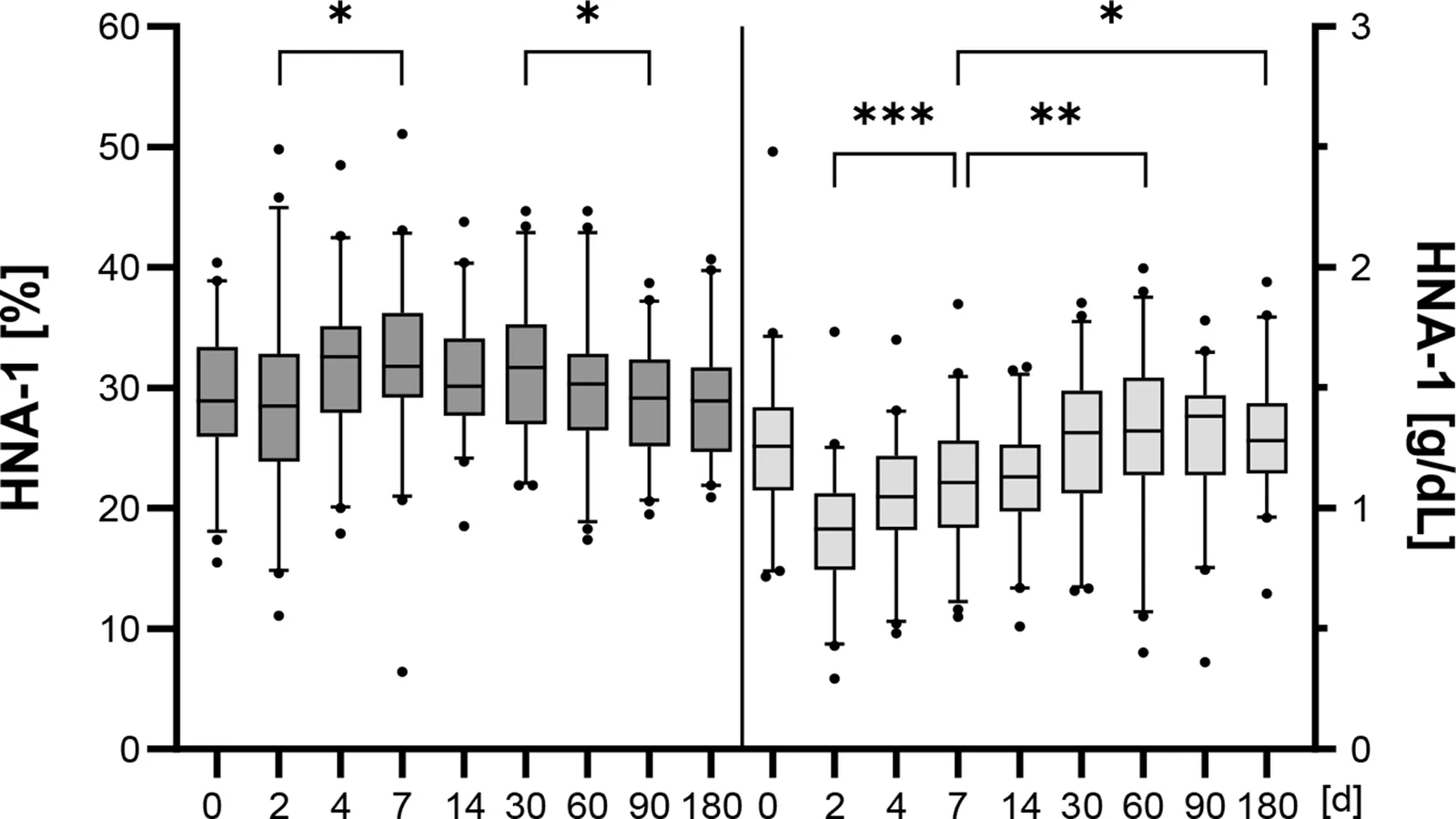
Human nonmercaptalbumin-1 after kidney transplantation. Shows the changes in human nonmercaptalbumin 1 (HNA-1) at 0 to 180 days after KTX with *n* = 42 patients in percentage (left y-axis) and total amount (right y-axis); the centre line indicates the median, the upper and lower limits represent the 25th and 75th percentiles, the whiskers the 5th-95th percentiles. Highest and lowest values are shown as dots. Asterisks mark differences between time points at a significance level of ** = *p* < 0.01 and *** = *p* < 0.001 with Residual Maximum Likelihood as a linear mixed-effects model (LMMs) and post-hoc Tukey-Kramer test

**Fig. 4:**
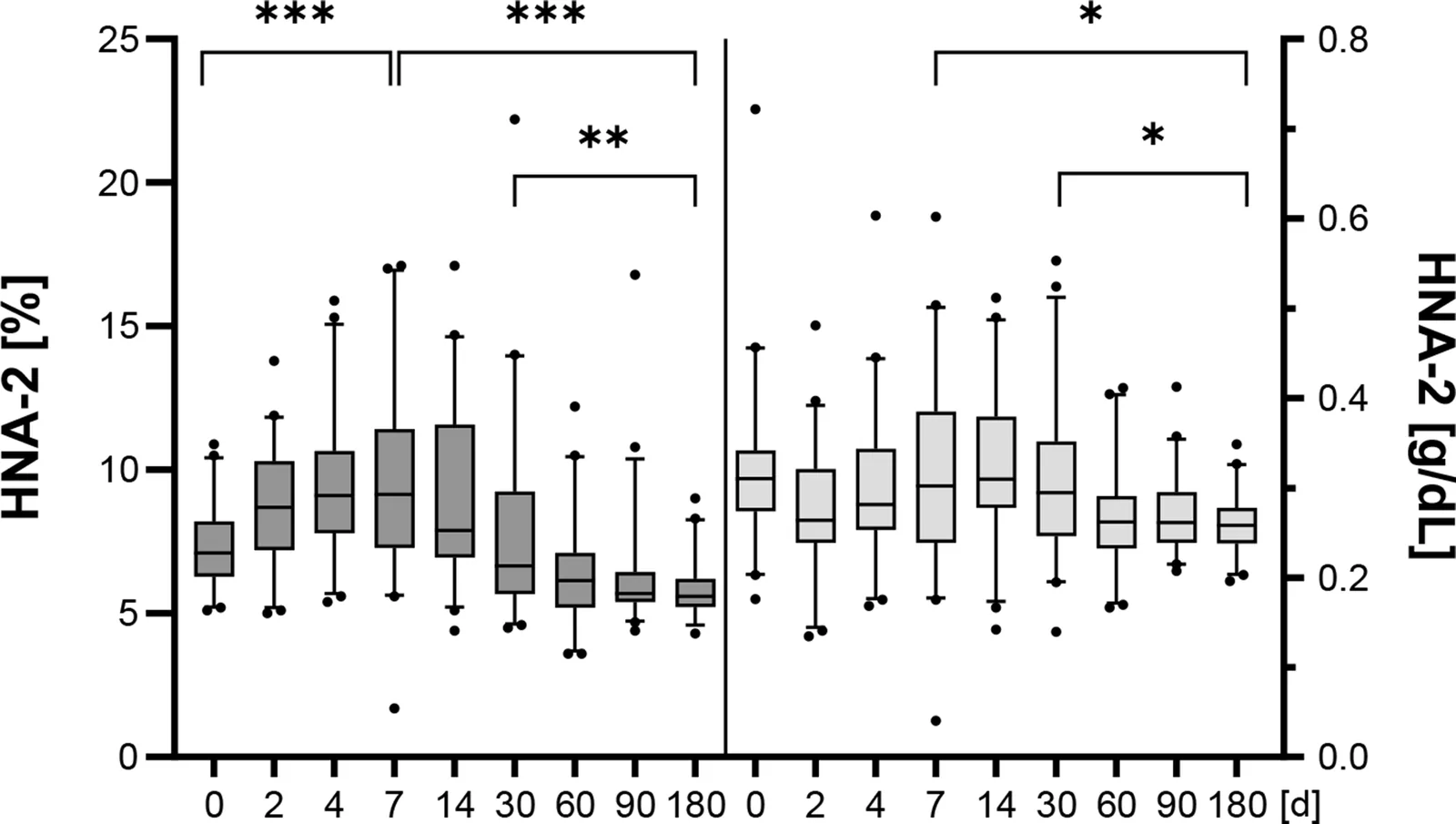
Human nonmercaptalbumin-2 after kidney transplantation. Shows the changes in human nonmercaptalbumin 2 (HNA-2) at 0 to 180 days after KTX with *n* = 42 patients in percentage (left y-axis) and total amount (right y-axis); the centre line indicates the median, the upper and lower limits represent the 25th and 75th percentiles, the whiskers the 5th-95th percentiles. Highest and lowest values are shown as dots. Asterisks mark differences between time points at a significance level of ** = *p* < 0.01 and *** = *p* < 0.001 with Residual Maximum Likelihood as a linear mixed-effects model (LMMs) and post-hoc Tukey-Kramer test. Additional significances could be shown between day 0 and 2 (*p* = 0.0001), 0 and 4 (*p* < 0.0001), 0 and 14 (*p* = 0.0016), 0 and 90 (*p* = 0.0146), 0 and 180 (*p* < 0.0001), 2 and 60, 90 and 180 (each *p* < 0.0001), 4 and 60, 90 and180 (each *p* < 0.0001), 7 and 60 and 90 (each *p* < 0.0001), 14 and 60, 90 and 180 (each *p* < 0.0001), 30 and 60 (*p* = 0.0101) and 30 and 90 (*p* = 0.0002) in percentage HNA-2. These further significances were not included in the figure for a clearer readability

**Fig. 5:**
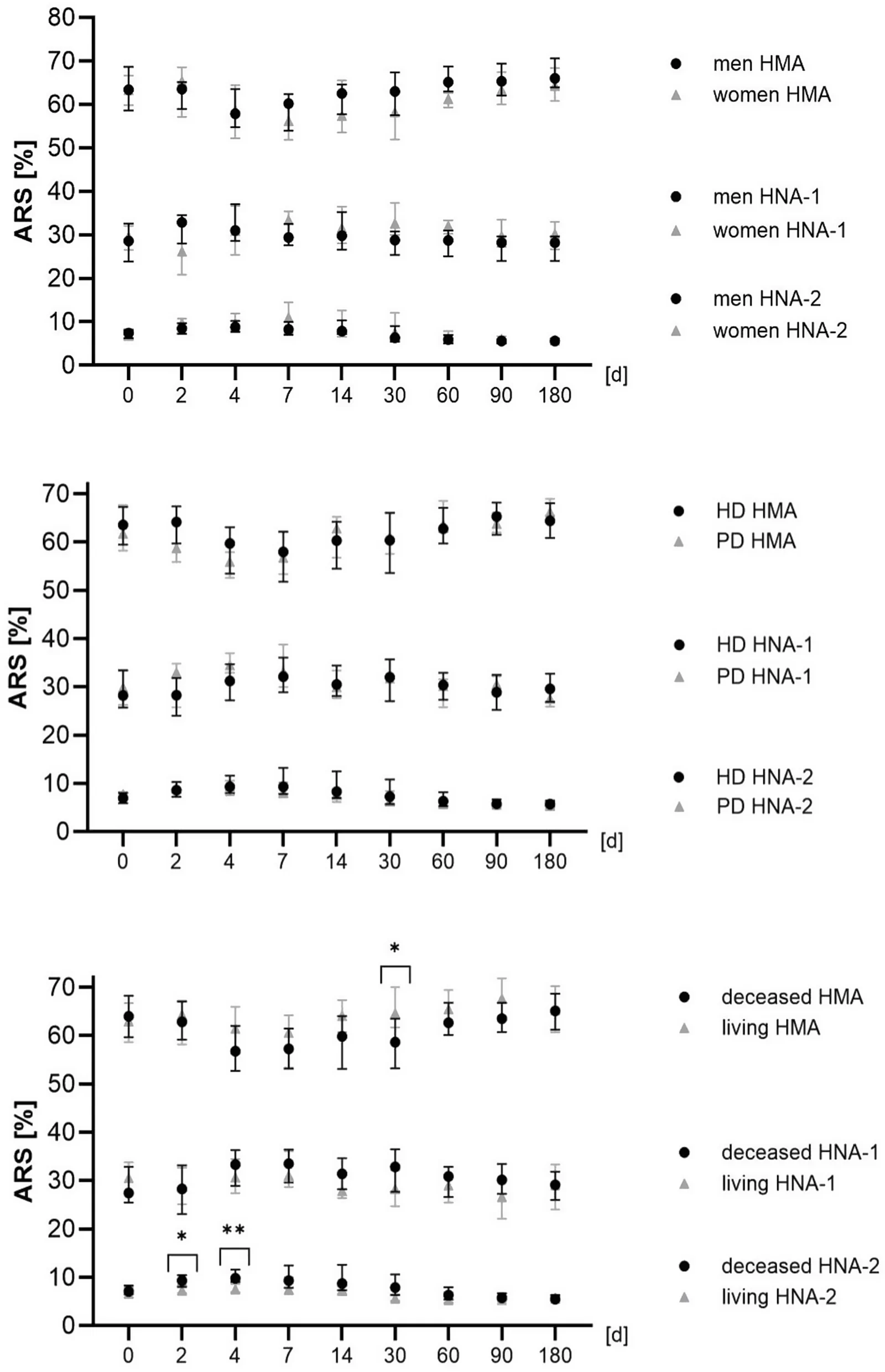
Alteration of albumin redox state up to 180 days after kidney transplantation in different subgroups. Shows the changes in human mercaptalbumin (HMA), human nonmercaptalbumin 1 (HNA-1) and human nonmercaptalbumin 2 (HNA-2) at 0 to 180 days after KTX with *n* = 42 patients broken down into different subgroups (men *n* = 26, women *n* = 16; HD *n* = 31, PD *n* = 8; deceased *n* = 28, living *n* = 14) as median and IQR. Asterisks mark differences between groups at a significance level of * = *p* < 0.05 and ** = *p* < 0.01 with Residual Maximum Likelihood as a linear mixed-effects model (LMMs) and post-hoc Tukey-Kramer test. Abbreviations: ARS albumin redox state, HD haemodialysis, PD peritoneal dialysis, KTX kidney transplantation, d days

In the early postoperative phase, former haemodialysis (HD) patients exhibited higher HMA levels than peritoneal dialysis (PD) patients (day 2: 64.2% [IQR 59.7–67.4%] vs. 58.9% [IQR 55.9–63.9%]). Although HMA declined more markedly in PD patients, recovery was greater by day 180 (HD: 64.4%; PD: 66.4%). Oxidized albumin fractions were initially higher in the PD subgroup, particularly on days 4 and 7 after KTX. Thereafter, oxidized fractions declined more rapidly in PD patients and remained below HD levels at day 180 (HNA-2: HD 5.7% [IQR 5.3–6.5%] vs. PD 5.4% [IQR 4.8–5.7%], *p* = 0.05). Additional data on ARS alterations in the overall cohort and according to sex, dialysis modality, and transplant type are provided in Table 2; Figs. 1, 2, 3, 4 and 5. However, it should be noted that these results are explorative comparisons without multiple adjusting due to the pilot character of the study.

**Table 2:** Albumin fractions at day 0, 30 and 180 after kidney transplantation

HMA				HNA-1			HNA-2		
Days after KTX	0d	30d	180d	0d	30d	180d	0d	30d	180d
**All** *n* = 42	63.5 60.0-67.2	61.3 56.2–66.3	65.3 61.2–69.9	28.9 26.0-33.4	31.7 27.0-35.3	28.9 24.7–31.7	7.1 6.3–8.2	6.7 5.7–9.3	5.6 5.3–6.2
Men *n* = 26	63.5 58.6–68.7	63.1 57.6–67.4	66.0 64.0-70.6	28.7 23.9–32.7	28.9 25.4–30.9	28.2 24.1–29.7	7.4 6.3–8.1	6.4 5.5-9.0	5.6 5.2–6.1
Women *n* = 16	63.4 59.8–66.7	58.3 52.0-62.7	64.4 60.8–68.4	29.7 26.6–32.1	32.7 30.0-37.4	30.3 26.7–33.0	6.9 6.1–8.4	7.7 6.0-12.1	5.8 5.3–6.2
**Transplant**									
Deceased *n* = 28	63.7 59.6–68.6	58.6 53.2–63.8	65.4 64.2–68.5	27.5 25.4–33	32.9 27.7–36.5	29.2 26.0-31.9	7.1 6.5–8.3	7.9 6.3–10.6	5.6 5.2–6.3
Living *n* = 14	63.1 58.6–66.8	65.0 61.9–70.7	64.3 60.1–70.6	30.7 27.2–33.8	28.4 24.7–32.1	29.1 24-33.3	6.9 5.8-8	5.7 5.1–6.5	5.7 5.5–6.1
**Pre-KTX Dialysis**									
haemodialysis *n* = 31	63.6 59.5–67.3	60.4 59.7–67.1	64.4 60.9–68.1	28.3 25.7–33.4	32.0 27.0-35.7	29.6 26.9–32.7	7.0 5.9-8.0	7.3 5.7–10.8	5.7 5.3–6.5
peritoneal *n* = 8	61.8 58.3–67.7	61.1 57.6–67.0	66.4 65.6–69.0	30.0 26.2–33.6	31.9 27.1–35.7	27.7 25.9–29.6	7.8 6.7–8.3	6.4 5.5–8.5	5.4 4.8–5.7

Table shows albumin fractions according to redox state (%) in median and interquartile range (IQR) before and on day 30 and 180 after kidney transplantation. The cohort (*n* = 42) is divided into men (*n* = 26) and women (*n* = 16), deceased (*n* = 28) and living (*n* = 14) transplantation and in pre-KTX maintenance haemo- (*n* = 31) or peritoneal (*n* = 9) dialysis. Abbreviations: human mercaptalbumin (HMA), human nonmercaptalbumin-1 (HNA-1), human non- mercaptalbumin-2 (HNA-2), day (d), kidney transplantation (KTX)

## Discussion

### Key findings

This is the first study to evaluate the effects of KTX and potential influencing factors on ARS in a European cohort. Prior to KTX, ARS showed a distinct shift toward oxidized fractions compared with the known distribution in healthy individuals, reflecting the effects of ESKD. This shift became even more pronounced immediately after KTX, primarily due to the loss of HMA. Over time, ARS recovered to pre-KTX levels and, in some cases, exceeded them. Remarkably, not all patients showed improvement beyond their pre-transplant ARS despite recovery of renal function by postoperative day 60. This observation indicates that restoration of kidney function alone may not fully explain postoperative ARS dynamics. Multiple factors associated with the perioperative and early post-transplant period, including surgical stress, ischaemia–reperfusion injury, inflammatory responses, delayed graft function, rejection, infections and treatment-related effects, may contribute to alterations in albumin redox state. However, the present study was not designed to determine the individual contribution of these factors.

Overall, this pilot study demonstrates that ARS undergoes dynamic changes following KTX, with recovery occurring over a different time course than restoration of renal function. These findings provide a rationale for further longitudinal studies investigating the determinants and clinical relevance of ARS changes after transplantation.

### Comparison with previous studies

Oxidative stress is a key feature of several diseases, and ARS has emerged as a sensitive marker of systemic redox imbalance. In liver cirrhosis, particularly acute-on-chronic liver failure, ARS shifts toward higher HNA-1 and HNA-2 fractions, with HNA-2 proposed as a potential biomarker [8, 12]. Similarly, CKD is associated with progressive albumin oxidation, as declining renal function correlates with increasing HNA-2 levels [13]. These findings are consistent with the markedly altered ARS observed before KTX in our cohort. Following transplantation, ARS gradually recovered, although the time course differed from that of renal function recovery. While kidney function improved rapidly in most patients, ARS remained altered in some individuals during follow-up. This temporal difference suggests that factors beyond kidney function may influence albumin redox balance after transplantation.

However, this observation should be interpreted cautiously. The early post-transplant period is characterised by multiple simultaneous processes that may affect ARS, including major surgery, ischaemia–reperfusion injury, perioperative inflammation, changes in albumin metabolism, albumin loss, delayed graft function, acute rejection, infectious complications and therapeutic interventions. The present study cannot distinguish the relative contribution of these factors, and persistent alteration of ARS should therefore not be interpreted as evidence of persistent systemic oxidative burden alone.

Experimental studies further demonstrate that ARS is modifiable, for example by dialysis membrane characteristics [14, 15]. Evidence on ARS after KTX remains limited. Tanaka et al. [16] reported partial recovery of reduced albumin fractions within 30 days after living-donor KTX in a Japanese cohort. In agreement with these findings, we observed recovery of ARS after KTX. However, our data additionally demonstrate an early postoperative decline in HMA, followed by gradual recovery during follow-up. Furthermore, not all patients exceeded their pre-KTX ARS values, highlighting variability in individual recovery patterns. Direct comparison between studies is limited by substantial methodological differences, including albumin isoform analysis and patient selection.

Alterations in ARS may also have functional implications, as albumin oxidation affects ligand-binding capacity and potentially alters the pharmacokinetics of immunosuppressive drugs [17, 18]. In addition, oxidized albumin may exert immunomodulatory effects [19]. Whether these mechanisms have clinical relevance in kidney transplant recipients remains unclear. Although altered ARS may theoretically influence albumin function and drug interactions, the present study does not allow conclusions regarding its association with graft dysfunction, rejection or immunosuppressive drug exposure. Further studies integrating ARS measurements with clinical outcomes and treatment data are needed.

Taken together, our findings indicate that ARS reflects dynamic changes during the early post-transplant period. However, these changes are likely influenced by multiple interacting factors, including recovery of kidney function, perioperative stress, inflammation, complications and treatment exposure. Our study extends current knowledge by demonstrating that ARS recovery may follow a different trajectory from restoration of renal function after KTX.

Although donor type, dialysis modality, postoperative complications and immunosuppressive treatment may influence ARS dynamics, the present cohort was not sufficiently powered to determine their independent effects. These factors should therefore be considered potential modifiers and important targets for future investigation rather than confirmed determinants of ARS changes.

### Strengths and limitations

This is the first European cohort study to investigate ARS alterations after KTX while accounting for dialysis modality and donor type. We analysed both reversible and irreversible albumin oxidation at the same molecular site and included recipients of both living and deceased donor kidneys. However, several limitations should be acknowledged. The study was conducted at a single centre and included a relatively small cohort, which limited statistical power, particularly for subgroup analyses and evaluation of potential determinants of ARS changes. It should also be noted that the subgroups were not formed by randomisation, and this has therefore led to differences in the baseline characteristics. In addition, postoperative complications resulted in incomplete datasets (one value at each of two points in time), further reducing the ability to assess longitudinal trajectories in specific clinical subgroups (Supplement Table 6). Due to the exploratory design and sample size, comprehensive adjustment for potential confounding factors was not feasible. Therefore, the present study cannot determine whether changes in ARS are primarily driven by recovery of kidney function or by other factors occurring during the early post-transplant period. In particular, clinically relevant variables including infectious complications, induction therapy, maintenance immunosuppression, corticosteroid exposure, rejection treatment and other perioperative interventions were not systematically evaluated in relation to ARS. These factors may influence oxidative stress, inflammation, albumin metabolism or ARS indirectly through their effects on rejection and infection risk and should therefore be considered potential confounders in future studies. While descriptive approaches can provide initial insights, as noted by Fox et al. [20], their interpretability remains limited and does not allow causal inference. Moreover, ARS represents only one aspect of the complex biological response to oxidative and inflammatory stress. Integration with additional biomarkers of inflammation, oxidative damage and metabolic alterations may provide a more comprehensive understanding of post-transplant redox regulation. Future multicentre studies with larger cohorts and expanded biomarker panels are therefore required to validate and extend these findings.

### Future prospects

This study highlights several directions for future research. Longitudinal assessment of ARS may provide additional insights into the biological processes occurring during the early period after KTX. However, whether ARS can serve as a clinically useful marker for monitoring transplant recipients or identifying specific complications remains to be established. The observation that some patients showed persistent alterations in ARS despite recovery of renal function warrants further investigation. Future studies should determine whether these differences reflect variations in perioperative stress responses, inflammation, immunosuppressive exposure, infectious complications or other clinical factors, and whether they are associated with relevant outcomes such as graft function, cardiovascular risk or long-term patient outcomes. Furthermore, investigating other post-translational modifications (e.g., glycation) together with the determination of ligands and detailed binding analyses could provide additional insights into altered albumin binding and transport function, particularly in patients with metabolic comorbidities such as diabetes or prediabetes. Given that ARS can be measured using a standardized HPLC-based approach, it may represent a promising research tool for future studies of post-transplant redox biology. Before clinical implementation, however, prospective multicentre studies with larger cohorts, longer follow-up and integration of clinical variables, treatment exposure and complementary biomarkers are required.

## Conclusion

This pilot study provides initial evidence from a European cohort on longitudinal changes in ARS following KTX. While renal function recovered rapidly after transplantation, ARS followed a different time course, with gradual recovery during follow-up and persistent alterations in some patients. These findings suggest that postoperative ARS dynamics are not determined by kidney function alone and may reflect the combined influence of multiple factors, including perioperative stress, inflammation, clinical complications and treatment-related exposures. Given the exploratory study design, larger prospective studies are required to determine the determinants and clinical relevance of ARS changes after KTX.

## Supplementary Information

Below is the link to the electronic supplementary material.


Supplementary Material 1: Supplement Table 1: Laboratory measurements



Supplementary Material 2: Supplement Table 2: Albumin quantity



Supplementary Material 3: Supplement Table 3: Course of inflammatory markers 0 up to 180 days after kidney transplantation



Supplementary Material 4: Supplement Table 4: Detailed information on patients with rejections



Supplementary Material 5: Supplement Table 5: Albumin redox state in patients with and without rejection



Supplementary Material 6: Supplement Table 6: Number of available patients at each time point


## Data Availability

The data underlying this article will be shared on reasonable request to the corresponding author.

## Abbreviations

AAV: Anti-neutrophil cytoplasmic antibody (ANCA)-associated vasculitis
ABMR: Antibody-mediated-rejection
ACLF: Acute on chronic liver failure
ADPKD: Autosomal dominant polycystic kidney disease
ALT: Alanine aminotransferase
ARS: Albumin redox state
AST: Aspartate aminotransferase
BUN: Blood urea nitrogen
CTA: Cellulose triacetate
DD: Deceased donation
ESKD: End-stage kidney disease
FFP: Fresh frozen plasma
Hb: Haemoglobin
HbA1c: Glycated haemoglobin A1c
HD: Haemodialysis, maintenance dialysis
HMA: Human mercaptalbumin
HNA-1: Human nonmercaptalbumin-1
HNA-2: Human nonmercaptalbumin-2
HPLC: High-performance liquid chromatography
HR: Hazard ratio
hsCRP: High sensitivity C-reactive protein
IL-6: INTERLEUKINE-6
KTX: Kidney transplantation
LDH: Lactate dehydrogenase
LMWH: Low molecular weight heparin
PCT: Procalcitonin
PMMA: Polymethylmethacrylate
RNS: Reactive nitrogen species
ROS: Reactive oxygen species
SLE: Systemic lupus erythematosus
TCMR: T-cell mediated rejection
TX: Transplantation
WBC: White blood cell count
