# Supplementary material for: Albumin redox status in the context of kidney transplantation – an observational, pilot study
Source: BMC Nephrol. 2026 Aug 28;27:511. doi: 10.1186/s12882-026-05316-y (PMC13525663; doi:10.1186/s12882-026-05316-y)
Supplement: Supplementary file 1 [file 12882_2026_5316_MOESM1_ESM.docx]

**Supplement Table 2. Albumin quantity**

|  | All n=42 | Deceased donation  n=28 | Living donation  n=14 | Maintenance dialysis  n=31 | Peritoneal dialysis  n=8 |
| --- | --- | --- | --- | --- | --- |
| Pre KTX | 4.4 | 4.2 | 4.5 | 4.3 | 4.1 |
|  | 4.1-4.7 | 3.9-4.7 | 4.4-4.7 | 4.1-4.7 | 3.8-4.7 |
| 2d post KTX | 3.3 | 3.2 | 3.6 | 3.3 | 3.1 |
|  | 3.1-3.7 | 3.1-3.5 | 3.3-3.9 | 3.2-3.8 | 2.9-3.4 |
| 4d post KTX | 3.3 | 3.3 | 3.4 | 3.3 | 3.4 |
|  | 3.2-3.6 | 3.1-3.6 | 3.3-3.9 | 3.2-3.6 | 3-3.6 |
| 7d post KTX | 3.5 | 3.4 | 3.6 | 3.5 | 3.4 |
|  | 3.2-3.7 | 3-3.6 | 3.4-3.9 | 3-3.7 | 3.1-3.7 |
| 14d post KTX | 3.9 | 3.7 | 4 | 3.7 | 4 |
|  | 3.6-4.1 | 3.5-4 | 3.8-4.3 | 3.4-4 | 3.8-4.1 |
| 30d post KTX | 4.3 | 4.3 | 4.5 | 4.3 | 4.3 |
|  | 3.9-4.5 | 3.9-4.4 | 3.8-4.6 | 3.7-4.5 | 4-4.8 |
| 60d post KTX | 4.4 | 4.4 | 4.6 | 4.4 | 4.6 |
|  | 4.2-4.7 | 4.2-4.7 | 4.3-4.8 | 4-4.7 | 3.4-5 |
| 90d post KTX | 4.5 | 4.5 | 4.6 | 4.5 | 4.7 |
|  | 4.3-4.8 | 4.3-4.7 | 4.3-4.9 | 4.3-4.8 | 4.4-4.9 |
| 180d post KTX | 4.6 | 4.5 | 4.6 | 4.5 | 4.7 |
|  | 4.3-4.8 | 4.3-4.8 | 4.4-4.9 | 4.3-4.8 | 4.3-4.9 |

Table shows median and interquartile range (IQR) of albumin quantity in g/dl in all (n=42) patients, in patients who received deceased (n=28) or living (n=14) transplant and in patients who underwent maintenance (n=31) or peritoneal (n=8) dialysis treatment before kidney transplantation from 0-180 days after KTX

Abbreviation: Interquartile range (IQR), Day (d), Kidney transplantation (KTX)

Regarding the total cohort significances could be shown between day 0 and 2, 4, 7 and 14 (each p<0.0001), 2 and 14 (p=0.0117), 2 and 30, 90 and 180 (each p<0.0001), 4 and 30, 60, 90 and 180 (each p<0.0001), 7 and 30, 60, 90 and 180 (each p<0.0001), 14 and 30 (p<0.05), 14 and 60, 90 and 180 (each p<0.0001) as well as between day 30 and 180 (p=0.0244).

Significant differences between the deceased and living donation subgroups could be shown at day 0, 2 and 14 with p < 0.05.

A significant difference between the maintenance and peritoneal dialysis subgroup could be shown at day 2 with p = 0.0011.
