# Supplementary material for: Albumin redox status in the context of kidney transplantation – an observational, pilot study
Source: BMC Nephrol. 2026 Aug 28;27:511. doi: 10.1186/s12882-026-05316-y (PMC13525663; doi:10.1186/s12882-026-05316-y)
Supplement: Supplementary file 2 [file 12882_2026_5316_MOESM2_ESM.docx]

**Supplement Table 1. Laboratory measurements**

| **Laboratory measurement** | **Baseline** | **Day 30 after KTX** | **Day 180 after KTX** |
| --- | --- | --- | --- |
| **WBC [× 10^9^/l]**  (normal, 3.6-9.2) | 6.5  (6-7.8) | 7.2  (6.2-9.3) | 6.2  (5.1-9.1) |
| **Hb [g/dl]**  (normal, 13.7-17.7) | 12.3  (11.1-13.6) | 11.1  (10.0-12.0) | 12.7  (11.4-13.7) |
| **Hct [l/l]**  (normal, 0.4-0.5) | 0.36  (0.31-0.40) | 0.34  (0.30-0.36) | 0.38  (0.35-0.42) |
| **PLT [× 10^9^/l]**  (normal, 140-320) | 231  (185.5-282.5) | 218  (194.3-286.3) | 240  (196-268.8) |
| **S-creatinine enzymatic [mg/dl]**  (normal 0.6-1.2) | 7  (5.5-9.6) | 1.5  (1.2-1.7) | 1.3  (1.1-1.6) |
| **eGFR (CKD-EPI, Cr) [ml/min/1.73m²]**  (normal >60) | 8  (5.8-9.6) | 54.9  (45.3-67.2) | 58.9  (49.6-72.0) |
| **eGFR (CKD-EPI, CysC) [ml/min/1.73 m²]**  (normal >60) | 8.7  (6.8-10.7) | 36.5  (29.5-42.0) | 44.7  (34.7-52.3) |
| **eGFR (CKD-EPI, Cr-CysC) [ml/min/1.73 m²]**  (normal >60) | 7.7  (5.8-9.0) | 43.0  (34.7-53.1) | 49.8  (39.9-60.2) |

| **Albuminuria [mg/g creatinine]**  (normal <30) | 1064  (326.9-1855) | 46.1  (23.7-97.0) | 18.1  (10.1-62.2) |
| --- | --- | --- | --- |
| **Cystatin C [mg/dl]**  (normal 0.5- 0.96) | 5.3  (4.5-6.3) | 1.8  (1.6-2.2) | 1.6  (1.4-1.8) |
| **HbA1c [%]**  (normal <5.7) | 5.4  (5.0-5.7) | 5.1  (4.7-5.4) | 5.6  (5.4-5.9) |
| **Glucose [mg/dl]**  (normal 70-100) | 86.0  (79.3-95.3) | 96.0  (84.0-104.0) | 93.5  (86.0-106.3) |
| **GOT [U/l]**  (normal <50) | 13.0  (10.5-18.5) | 13.0  (11.8-17.8) | 16.0  (14.0-21.5) |
| **GPT [U/l]**  (normal < 50) | 19.0  (14.5-32.0) | 26.0  (17.0-37.0) | 24.0  (18.8-34.3) |
| **Bilirubin [mg/dl]**  (normal <1.1) | 0.3  (0.2-0.4) | 0.4  (0.3-0.4) | 0.5  (0.33-0.6) |
| **CRP-sens. [mg/l]**  (normal <1.0) | 0.19  (0.08-0.44) | 0.16  (0.02-0.47) | 0.12  (0.04-0.44) |
| **IL-6 [pg/ml]**  (normal <15) | 2.7  (2.7-3) | 2.7  (2.7-3.7) | 2.7  (2.7-3.3) |
| **Serum-potassium [g/dl]**  (normal 3.5-5.0) | 5.3  (4.8-5.8) | 4.5  (4.3-4.9) | 4.3  (4.1-4.6) |

Laboratory parameters of the study cohort included hematologic values, renal function markers, metabolic indicators, and inflammatory markers before (baseline) versus 30 and 180 days after kidney transplantation. Data are reported as median and interquartile range (IQR), with reference ranges provided in parentheses.

Abbreviations: Kidney transplantation (KTX), White blood cell count (WBC), Haemoglobin (Hb), Hematocrit (Hct), Platelet count (PLT), Estimated glomerular filtration rate (eGFR), Chronic Kidney Disease Epidemiology Collaboration equation (CKD-EPI), Creatinine-based (Cr), Cystatin C–based (CysC), Combined creatinine and cystatin C formula (Cr-CysC), Serum creatinine (S-Creatinine), Glycated haemoglobin (HbA1c), Glutamate oxaloacetate transaminase (GOT), Glutamate pyruvate transaminase (GPT) and High-sensitivity C-reactive protein (CRP-sens.), Interleukine-6 (IL-6)
