# Supplementary material for: Albumin redox status in the context of kidney transplantation – an observational, pilot study
Source: BMC Nephrol. 2026 Aug 28;27:511. doi: 10.1186/s12882-026-05316-y (PMC13525663; doi:10.1186/s12882-026-05316-y)
Supplement: Supplementary file 3 [file 12882_2026_5316_MOESM3_ESM.docx]

**Supplement Table 5 albumin redox state in patients with and without rejection**

|  | **HMA** | | | **HNA-1** | | | **HNA-2** | |
| --- | --- | --- | --- | --- | --- | --- | --- | --- |
|  | rejection | | non-rejection | rejection | non-rejection | | rejection | non-rejection |
|  |  | |  |  |  | |  |  |
| **Pre KTX** | 58.9  56.5-70.0 | 63.5  59.6-67.1 | | 30.4  23.9-34.6 | | 28.9  26.1-32.9 | 6.7  5.8-8.9 | 7.3  6.5-8.2 |
|  |  |  | |  | |  |  |  |
| **2d**  **post KTX** | 59.3  47.8-61.1 | 64.2  58.9-67.4 | | 32.3  25.7-42.5 | | 28.3  23.7-32.5 | 8.3  7.1-9.7 | 8.8  7.2-10.4 |
|  |  |  | |  | |  |  |  |
| **4d**  **post KTX** | 56.9  50.1-64.8 | 57.9  53.5-64.0 | | 32.9  28.4-40.9 | | 31.2  27.9-34.7 | 9.2  7.5-9.8 | 9.1  7.9-11.4 |
|  |  |  | |  | |  |  |  |
| **7d**  **post KTX** | 56.6  48.9-61.9 | 58.5  53.5-62.0 | | 33.8  29.9-43.0 | | 31.8  29.0-35.5 | 8.4  6.6-9.6 | 9.3  7.3-12.5 |
|  |  |  | |  | |  |  |  |
| **14d**  **post KTX** | 51.2  47.5-63.3 | 61.9  56.5-64.9 | | 30.1  28.1-40.4 | | 30.2  27.5-33.8 | 10.4  6.1-14.0 | 7.9  7.0-11.2 |
|  |  |  | |  | |  |  |  |
| **30d**  **post KTX** | 58.8  50.2-65.8 | 61.3  57.1-66.6 | | 29.3  23.3-37.2 | | 32.0  27.0-35.1 | 9.1  7.3-15.2 | 6.4  5.6-9.0 |
|  |  |  | |  | |  |  |  |
| **60d**  **post KTX** | 63.7  57.8-66.5 | 63.6  61.0-68.2 | | 28.8  25.4-33.9 | | 30.5  26.6-32.8 | 8.2  6.4-10.1 | 6.0  5.1-6.5 |
|  |  |  | |  | |  |  |  |
| **90d**  **post KTX** | 61.9  60.1-66.8 | 65.2  61.7-69.4 | | 29.5  26.3-34.2 | | 29.2  25.1-32.2 | 6.6  5.6-10.2 | 5.6  5.3-6.2 |
|  |  |  | |  | |  |  |  |
| **180d**  **post KTX** | 68.40  56.6-71.2 | 65.3  61.8-69.9 | | 27.8  23.3-37.9 | | 28.9  24.7-31.7 | 6.3  4.5-8.6 | 5.6  5.3-6.2 |
|  |  |  | |  | |  |  |  |

Table shows albumin fractions according to redox state (%) in median and interquartile range (IQR) before and up to 180 days after kidney transplantation. The cohort (n=42) is divided into patients with rejection (n=6) and patients without rejection (n=36). Abbreviations: human mercaptalbumin (HMA), human nonmercaptalbumin-1 (HNA-1), human non- mercaptalbumin-2 (HNA-2), day (d), kidney transplantation (KTX)
