# Supplementary material for: Albumin redox status in the context of kidney transplantation – an observational, pilot study
Source: BMC Nephrol. 2026 Aug 28;27:511. doi: 10.1186/s12882-026-05316-y (PMC13525663; doi:10.1186/s12882-026-05316-y)
Supplement: Supplementary file 4 [file 12882_2026_5316_MOESM4_ESM.docx]

**Supplement Table 4 Detailed information on patients with rejections**

|  | **Patient 1** | **Patient 2** | **Patient 3** | **Patient 4** | **Patient 5** | **Patient 6** |
| --- | --- | --- | --- | --- | --- | --- |
| **Sex** | M | W | M | M | M | m |
| **Age** | 18 | 31 | 44 | 45 | 50 | 60 |
| **Dialysis mode** | HD | HD | HD | PD | PD | HD |
| **Graft type** | LD | DD | DD | DD | DD | DD |
| **Time of biopsy (d after KTX)** | 4 | 4 | 10 | 7 | 7 | 5 |
| **BANFF category** | 4 I A | 4 II A + 6 | 2 + 6 | 3 | 3 + 6 | 4 II A + 6 |
| **DSA** | no | no | yes | no | no | no |
| **Rejection treatment** | Intravenous cortisone for 3 days | Intravenous cortisone for 3 days, followed by thymoglobulin | Plasmapheresis with fresh frozen plasma from day 11 to day 17 for five times, followed by intravenous immunoglobulin for 3 days | Intravenous cortisone for 3 days | Intravenous cortisone for 3 days | thymoglobulin followed by intravenous immunoglobulin |
| **Graft function 180 days after KTX** | CKD G4 T | CKD G5 T | CKD G4 T | CKD G3a T | CKD G3b T | CKD G5 T |
|  |  |  |  |  |  |  |
