# Supplementary material for: Albumin redox status in the context of kidney transplantation – an observational, pilot study
Source: BMC Nephrol. 2026 Aug 28;27:511. doi: 10.1186/s12882-026-05316-y (PMC13525663; doi:10.1186/s12882-026-05316-y)
Supplement: Supplementary file 5 [file 12882_2026_5316_MOESM5_ESM.docx]

**Supplement Table 3 Course of inflammatory markers 0 up to 180 days after kidney transplantation**

| Days after KTX | 0d | 2d | 4d | 7d | 14d | 30d | 60d | 90d | 180d |
| --- | --- | --- | --- | --- | --- | --- | --- | --- | --- |
| **hs CRP [mg/l]**  (normal <1.0) | 0.19  0.09-0.47 | 2.35  1.00-3.84 | 0.97  0.50-2.70 | 0.54  0.20-1.95 | 0.25  0.06-1.00 | 0.16  0.02-0.47 | 0.11  0.04-0.50 | 0.11  0.04-0.051 | 0.12  0.04-0.44 |
| **IL-6 [pg/ml]**  (normal <15) | 2.7  2.7-2.95 | 5.9  3.65-10.6 | 3.55  2.7-5.6 | 3.1  2.7-7.05 | 2.7  2.7-3.65 | 2.7  2.7-3.7 | 2.7  2.7-3.6 | 2.7  2.7-3.85 | 2.7  2.7-3.3 |

Table shows the course of high sensitive C-reactive protein (hs CRP) and interleukin 6 (IL-6) in

median and interquartile range (IQR) before and up to 180 days after kidney transplantation

(KTX) for n=42 patients.
