# Supplementary material for: Albumin redox status in the context of kidney transplantation – an observational, pilot study
Source: BMC Nephrol. 2026 Aug 28;27:511. doi: 10.1186/s12882-026-05316-y (PMC13525663; doi:10.1186/s12882-026-05316-y)
Supplement: Supplementary file 6 [file 12882_2026_5316_MOESM6_ESM.docx]

Supplement Table 6: Number of available patients at each time point

| **time point** | **all** | **m** | **w** | **HD** | **PD** | **rejection** | **non-rejection** |
| --- | --- | --- | --- | --- | --- | --- | --- |
| 0 | 42 | 26 | 16 | 31 | 8 | 6 | 36 |
| 2 | 42 | 26 | 16 | 31 | 8 | 6 | 36 |
| 4 | 41 | 25 | 16 | 31 | 7 | 6 | 35 |
| 7 | 42 | 26 | 16 | 31 | 8 | 6 | 36 |
| 14 | 42 | 26 | 16 | 31 | 8 | 6 | 36 |
| 30 | 42 | 26 | 16 | 31 | 8 | 6 | 36 |
| 60 | 42 | 26 | 16 | 31 | 8 | 6 | 36 |
| 90 | 42 | 26 | 16 | 31 | 8 | 6 | 36 |
| 180 | 40 | 25 | 15 | 29 | 8 | 4 | 36 |
